# Engineering a novel endopeptidase based on SARS 3CL^pro^

**DOI:** 10.2144/000113303

**Published:** 2009-04-25

**Authors:** Chih-Jung Kuo, Yan-Ping Shih, Daphne Kan, Po-Huang Liang

**Affiliations:** 1 Institute of Biological Chemistry, Academia Sinica and Core Facility of Recombinant Protein Production, Taipei, Taiwan; 2 Institute of Biochemical Sciences, National Taiwan University, Taipei, Taiwan

**Keywords:** SARS-CoV, 3CL protease, endopeptidase, tag cleavage, parallel cloning

## Abstract

A 3C-like protease (3CL^pro^) from the severe acute respiratory syndrome–coronavirus (SARS-CoV) is required for viral replication, cleaving the replicase polyproteins at 11 sites with the conserved Gln↓(Ser, Ala, Gly) sequences. In this study, we developed a mutant 3CL^pro^ (T25G) with an expanded S1′ space that demonstrates 43.5-fold better *k*_cat_/K_*m*_ compared with wild-type in cleaving substrates with a larger Met at P1′ and is suitable for tag removal from recombinant fusion proteins. Two vectors for expressing fusion proteins with the T25G recognition site (Ala-Val-Leu-Gln↓Met) in *Escherichia coli* and yeast were constructed. Identical cloning sites were used in these vectors for parallel cloning. *Pst*I was chosen as a 5′ cloning site because it overlapped the nucleotide sequence encoding the protease site and avoided addition of extra amino acids at the N terminus of recombinant proteins. 3CL^pro^ (T25G) was found to have a 3-fold improvement over TEV^pro^ in tag cleavage at each respective preferred cleavage site.

## Introduction

Severe acute respiratory syndrome–coronavirus (SARS-CoV) caused an outbreak in 2003 that killed approximately 800 patients worldwide (1). A 3C-like protease from the virus, 3CL^pro^, is required to cleave 11 sites of the polyproteins pp1a (486 kDa) and pp1ab (790 kDa) for their maturation (2). 3CL^pro^ is a chymotrypsin-like protease, but it uses Cys as a nucleophile for catalysis (3). Analogous to 3C proteases of picornaviruses, 3CL^pro^ has substrate specificity in cleaving the amide bond between P1-Gln and a small amino acid such as Ser, Ala, or Gly at P1′ (4,5). As evident in its 3-D structure (6,7), this small P1′ residue is near Thr25, which likely determines the substrate specificity.

It was previously demonstrated that the recombinant SARS 3CL^pro^ can undergo auto-processing (7,8), which indicates its potential as a tag-cleavage endopeptidase. However, it would need to be capable of cleaving Q↓M, since Met is the most common first residue at protein N-termini. In this study, we replaced the 3CL^pro^ Thr25 with the smaller Gly residue to expand the S1′ site and found that the mutant protease cleaved peptides with larger amino acids such as Met at P1′ with high efficiency. The results presented here demonstrate that Thr25 is essential to determine P1′ substrate specificity and that the T25G mutant can be used as a novel endopeptidase for tag cleavage of recombinant fusion proteins in addition to the commonly used thrombin, Factor Xa (FXa), and tobacco etch virus protease (TEV^pro^). Moreover, we have constructed two vectors, using prokaryotic and eukaryotic hosts, which contained the nucleotides encoding the T25G recognition site AVLQ↓M between the tags and the N-terminal Met of the target proteins. In these vectors, *Pst*I (CTGCAG) was chosen as a 5′ cloning site, since its sequence overlapped the nucleotide sequence (GCGGTGCTGCAG) encoding the protease recognition site. Identical 5′-*Pst*I/3′-*Xho*I cloning sites in these vectors were used to allow sticky-end DNA fragments of the target genes generated by PCR (9) to ligate with these vectors simultaneously in a strategy called parallel cloning (10,11). These vectors, in conjunction with the T25G protease, provide new tools for convenient protein production in different hosts and tag cleavage to yield recombinant proteins with authentic sequences.

## Materials and methods

### Expression and purification of mutant 3CL^pro^

Expression and purification of wild-type and mutant SARS 3CL^pro^ in *Escherichia coli* was accomplished according to reported procedures (12). T25G and T25S mutants were prepared from the wild-type by using the QuickChange site-directed mutagenesis kit (Cat. no. 200518; Stratagene, La Jolla, CA, USA). C-terminally His-tagged T25G was expressed using pET16b vector (Cat. no. 69662; Novagen, Darmstadt, Germany).

### Construction of the expression vectors for producing tag-cleavable fusion proteins in *E. coli* and yeast

The UPPs-encoding gene (13) was employed as a template for PCR using primers containing the nucleotides encoding the T25G 3CL^pro^ recognition site AVLQ, and the TEV^pro^ recognition site EDLYFQ, respectively. The PCR products were purified from an agarose gel following electrophoresis and cloned into the pET32Xa/Lic vector (Novagen). To serve as a control, the UPPs fusion protein with AAAQ instead of AVLQ was also expressed.

For expressing EGFP fusion proteins in yeast, primers were used to generate a PCR product that was ligated into pHTPY7, which was modified from pPICZαA (Invitrogen) by incorporating nucleotides encoding a starch binding domain (SBD) (14) and AVLQ cleavage site.

### Evaluation of tag removal by the proteases

The UPPs and EGFP fusion proteins were purified using NiNTA columns. To examine the tag cleavage reactions, the purified fusion proteins (5.4 µM each) were treated with 0.1 µM wild-type and two mutant (T25G and T25S) 3CL^pro^ for 90 min at 37°C. For time course measurements, the fusion proteins (5.4 µM each) were treated with T25G (0.1 µM) at 37°C. The reactions were stopped by 2% trifluoroacetic acid after appropriate time periods and analyzed by SDS-PAGE. For comparing the tag cleavage efficiency of T25G and TEV^pro^ (Invitrogen), the fusion proteins (Tags-AVLQ-UPPS and Tags-ENLYFQ-UPPS, 5.4 µM each) were treated with 0.1 µM T25G and TEV^pro^ at 37°C, respectively and then analyzed by SDS-PAGE.

### Substrate specificity and kinetic parameters of the mutant SARS 3CL^pro^

The peptides used as substrates for the T25G protease were synthesized via solid phase, using a 433A peptide synthesizer (Applied Biosystems, Foster City, CA, USA). Each peptide (100 µM) was incubated with 0.1 µM T25G for 1, 2, and 6 h, and the subsequent mixtures were analyzed by HPLC on a C-18 reverse-phase analytic column. Cleavage products were resolved using a 30-min, 2–90% linear gradient of acetonitrile plus 0.1% TFA. The product peak areas were integrated to calculate the reaction rates for each peptide substrate. For *K*_m_ and *k*_cat_ measurements, 0.1 µM T25G and 10–200 µM SAVLQ↓MGFRK substrate were used, and the plot of initial rates within 10% substrate consumption versus different substrate concentrations was fitted to the Michaeli-Menten equation using the KaleidaGraph computer program (Synergy Software, Reading, PA, USA).

## Results and discussion

### Design, preparation, and characterization of T25G and T25S 3CL^pro^

**Figure 1.**
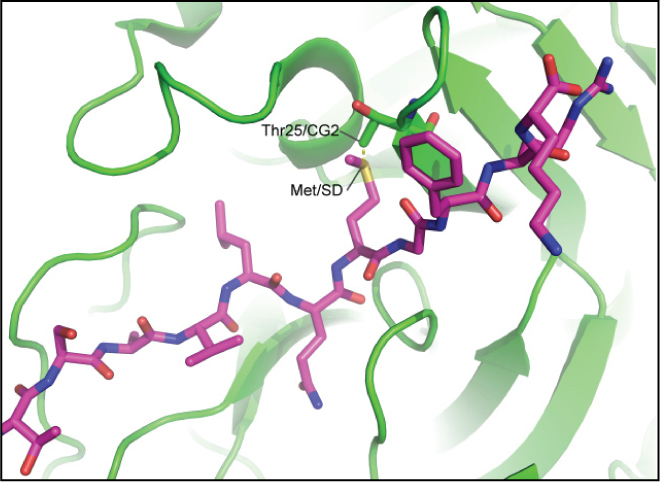
Structural basis for T25 mutation. Predicted structural model of SARS-CoV 3CL^pro^ with a modified peptide containing Met at P1′ (Thr-Ser-Ala-Val-Leu-Gln-Met-Phe-Arg-Lys), based on the crystal structure of SARS-CoV 3CL^pro^ H41A mutant in complex with a peptide (PDB entry: 2Q6G).

Based on the crystal structure of SARS-CoV 3CL^pro^ in complex with a peptide (Protein Data Bank entry 2Q6G; www.rcsb.org/pdb), we have generated a structural model of the protease binding with a modified peptide (Thr-Ser-Ala-Val-Leu-Gln-Met*-Phe-Arg-Lys), where the Ser at P1′ was changed to Met (indicated by the asterisk). We found that Thr25/CG2 of the 3CL^pro^ is within a short distance of 1.32 Å of Met/SD of the peptide (Figure 1) as determined by the COOT program (15). Thus, Thr25 may be replaced by a smaller Gly or Ser (maintaining an -OH group) for better accommodation of Met at P1′.

**Figure 2.**
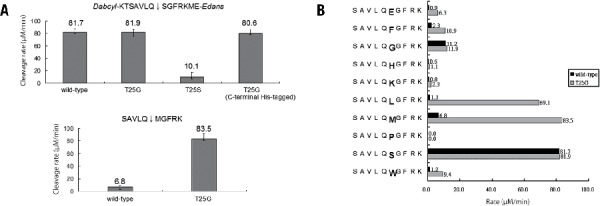
Characterization of the wild-type and mutant 3CL^pro^. (A) Activities of wild-type, T25G, T25S, and C-terminal His-tagged T25G 3CL^pro^ against a fluorogenic substrate Dabcyl-KTSAVLQSGFRKME-Edans (upper panel). The cleavage rate by T25G 3CL^pro^ (83.5 µM/min) was remarkably higher than the rate of the wild-type (6.8 µM/min) against a peptide substrate SAVLQ↓MGFRK containing Met at P1′ (lower panel). (B) Substrate specificity of T25G 3CL^pro^ at P1′ site. Activities of wild-type and T25G 3CL^pro^ using 10 peptides SAVLQXGFRK (X = Glu, Phe, Gly, His, Lys, Leu, Met, Pro, Ser, and Trp) as substrates.

To test the above hypothesis, T25G and T25S 3CL^pro^ were expressed in *E. coli* and purified using NiNTA chromatography. Yields were approximately 20 mg/L medium, which were similar to yields from wild-type preparations. T25G, with a C-terminal His-tag, was also prepared for removing the protease using NiNTA after tag cleavage. The enzymatic activities of these protease forms were measured using the fluorogenic substrate Dabcyl-KTSAVLQSGFRKME-Edans, as described previously (12). Compared with the activity of wild-type, no significant difference was observed for T25G and C-terminal His-tagged T25G, but T25S showed almost complete loss of activity (Figure 2A, upper panel). However, for the peptide substrate SAVLQ↓MGFRK containing Met at P1′, T25G showed significantly higher specific activity than the wild-type (83.5 µM/min versus 6.8 µM/min) (Figure 2A, lower panel), indicating that T25G can tolerate the larger residue Met at P1′. In comparison with the *k*_cat_ of 1.6 ± 0.2 min^−1^ and the *K*_m_ of 76.6 ± 3.5 µM for the wild-type, the T25G mutant displayed the *k*_cat_ of 16.2 ± 0.5 min^−1^ and the *K*_m_ of 18.6 ± 2.4 µM (43.5-fold higher *k*_cat_/*K*_m_) against the SAVLQ↓MGFRK substrate.

### Substrate specificity and kinetic parameters of the mutant SARS 3CL^pro^

Next, peptides corresponding to the N-terminal maturation site of SARS 3CL^pro^ with 10 selected variations (Glu, Phe, Gly, His, Lys, Leu, Met, Pro, Ser, and Trp) at P1′ were prepared and used to evaluate the substrate specificity of T25G. As shown in Figure 2B, T25G showed a 12-fold and 8-fold higher activity against the substrates with Met and Leu at P1′, respectively. For the optimal substrate SAVLQ↓SGFRK of the wild-type, T25G mutant showed about equal activity, indicating that T25G still holds the P1′ residue of the small side chain. Similar to the wild-type, T25G did not tolerate peptides with bulky amino acids such as Trp and Phe orcharged amino acids such as Glu and Lys. The peptide with P1′-Pro showed no activity.

### Construction of *E. coli* and yeast vectors to express tag-cleavable fusion proteins by T25G 3CL^pro^

**Figure 3.**
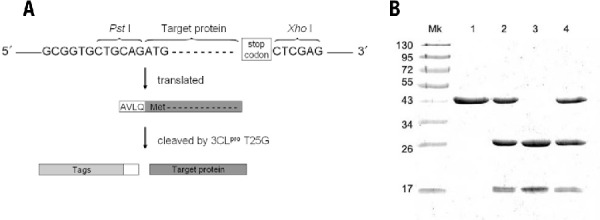
**Expression of the UPPs fusion protein in *E. coli* and tag cleavage by the wild-type and mutant 3CL^pro^**. (A) The strategy of using *Pst*I as a 5′ cloning site (CTGCAG) that is part of the sequence GCGGTGCTGCAG encoding the protease recognition site is illustrated. (B) 5.4 µM UPPs fusion protein before treatment (lane 1) and after treatment with 0.1 µM wild-type (lane 2), T25G (lane 3), and T25S 3CL^pro^ (lane 4) for 90 min at 37°C are shown. Only T25G can efficiently cleave the fusion protein to generate tag-free UPPs.

We constructed two vectors for use with *E. coli* and yeast to express fusion proteins with an AVLQ recognition site to test tag cleavage by T25G. The *Pst*I site CTGCAG, which is part of the AVLQ-encoding sequence GCGGTGCTGCAG, was used as a 5′ cloning site, in conjunction with the 3′ *Xho*I site, for sticky-end ligation with the PCR product of the target gene (see the strategy illustrated in Figure 3A). As shown in Figure 3B, the purified 5.4 µM fusion UPPs (lane 1) was incubated with 0.1 µM wild-type and two mutant 3CL^pro^ (T25G and T25S), and the final products are shown in lanes 2, 3, and 4, respectively. Only T25G mutant efficiently cleaved the fusion protein, yielding tag-free UPPs (28.3 kDa) and the tags (17.6 kDa) as shown in lane 3. Under these conditions, the tag cleavage reaction was completed <90 min (data not shown). As a control experiment, a UPPs fusion protein with an AAAQ sequence was incubated with the protease, but was not cleaved (data not shown), indicating that T25G specifically recognized the AVLQ cleavage site.

Using a yeast *Pichia* expression system, the EGFP fusion protein with SBD, His-tag, and AVLQ site was overexpressed. SBD was included for the purpose of using starch as an affinity matrix for protein purification, which would lower the associated costs. With 0.1 µM T25G, the cleavage of the fusion protein (5.4 µM) was completed <120 min (data not shown).

### Comparison of tag cleavage using TEV protease and T25G 3CL^pro^

Since TEV^pro^ is one of the most commonly used endopeptidases for tag cleavage and shares a similar substrate specificity with SARS 3CL^pro^, we compared the efficiency of tag cleavage using T25G 3CL^pro^ to that of TEV^pro^ against the *E. coli*-expressed UPPs fusion proteins containing their preferred recognition sites. Compared with TEV^pro^, T25G showed a 3-fold higher cleavage rate (0.106 µM/min versus 0.035 µM/min; data not shown). However, compared with that of FXa, another commonly used endopeptidase, the cleavage rate of T25G was 1.7 times lower (0.106 µM/min versus 0.178 µM/min; data not shown). TEV^pro^ generally accepts any amino acid at P1′ except Pro (16). However, besides Met, T25G prefers small residues that are actually very common N-terminal residues of “native proteins” due to the post-translational action of Met amino peptidase. This suggests a great advantage of using T25G as a novel endopeptidase for tag removal. These engineered vectors and T25G can be assembled as a kit for the maximal production of soluble and functional proteins with authentic sequences.
